# Efficacy of Different Protocols of Radioiodine Therapy for Treatment of Toxic Nodular Goiter: Systematic Review and Meta-Analysis of the Literature

**DOI:** 10.5812/ijem.14424

**Published:** 2014-04-01

**Authors:** Haleh Rokni, Ramin Sadeghi, Zohreh Moossavi, Giorgio Treglia, Seyed Rasoul Zakavi

**Affiliations:** 1 Endocrinology Research Center, Mashhad University of Medical Sciences, Mashhad, IR Iran; 2 Nuclear Medicine Research Center, Mashhad University of Medical Sciences, Mashhad, IR Iran; 3 Department of Nuclear Medicine and PET/CT Centre, Oncology Institute of Southern Switzerland, Bellinzona, Switzerland

**Keywords:** Thyrotoxicosis, Goiter, Nodular, Iodine, Hyperthyroidism

## Abstract

**Context:**

To evaluate treatment success and hypothyroidism following main methods of radioiodine therapy of toxic nodular goiter (TNG); calculated versus fixed dose and high versus low dose of radioiodine.

**Evidence Acquisitions:**

We searched MEDLINE and SCOPUS databases from inception till July 2013, for clinical trials that compared two different methods of radioiodine administration in TNG. The trials were classified into two groups, those that compared fixed versus calculated dosimetry method and those that assessed high fixed dose versus low fixed dose method. Treatment response was defined as euthyroidism or hypothyroidism, one year after radioiodine administration. We calculated the risk ratio and risk difference of treatment response as well as permanent hypothyroidism as outcome variables. Random effects model was used for data pooling.

**Results:**

The literature search yielded 2538 articles. Two randomized and five non-randomized clinical trials with 669 patients met the eligibility criteria for the meta-analysis. Patients with TNG who were treated according to the calculated method had 9.6% higher cure rate (risk ratio=1.17) and only 0.3% more permanent hypothyroidism compared to patients treated with the fixed dose method. There was no significant difference in the amount of administered radio-iodine in the two groups. Patients treated with fixed high dose had 18.1% more cure rate (risk ratio = 1.2) and 23.9% more permanent hypothyroidism (risk ratio = 2.40) compared to patients treated by fixed low dose protocols.

**Conclusions:**

Calculated radioiodine therapy may be preferred to fixed dose method in patients with TNG. High dose methods are associated with more response and more hypothyroidism.

## 1. Context

Hyperthyroidism is a common disorder throughout the world and graves’ disease and toxic nodular goiter (TNG) are the most common causes of hyperthyroidism (1). Although most physicians consider radioiodine as the first line of treatment for TNG, there is no consensus regarding the optimum dose of radioiodine (I-131) (2).

Generally, four different strategies for radioiodine treatment of hyperthyroidism can be identified in the literature; fixed high dose (FHD), fixed low dose (FLD), calculated high dose (CHD) and calculated low dose (CLD). Most studies reported the result of I-131 therapy using only one of these methods (3). A few studies have compared two different methods of I-131 therapy in a clinical trial (4, 5). Fixed dose method is the simplest method and effective in treatment of patients with TNG, however there is no strong correlation between administered dose and delivered dose to thyroid (6, 7). A survival study for fixed dose treatment showed that only 13% of patients with toxic adenoma who received 555 MBq of I-131 (low dose) remained hyperthyroid within one year after this dose (8) while another study found similar results with higher doses of I-131 (888-1110 MBq) (9). Comparison of these two articles suggests that a significant number of patients in the second study may be over treated (10). Treatment of hyperthyroidism with high dose of I-131 causes early cure of hyperthyroidism in majority of patients, however this is associated with higher rate of hypothyroidism as well (5). Low dose of I-131, on the other hand, may result in delayed improvement of hyperthyroidism and the patient will be at risk of cardiovascular complication (11). The aim of this systemic review and meta-analysis was to compare treatment success as well as rate of hypothyroidism of fixed versus calculated dose and high versus low dose I-131 therapy in patients with TNG. 

## 2. Evidence Acquisition

We searched Medline and Scopus from their inception till end of July 2013 for clinical trials that compared two different methods of I-131 administration in TNG. The search term consisted of “(thyrotoxicosis OR hyperthyroid OR toxic OR hot OR Plummer) AND (adenoma OR Nodul*) AND (iodine OR 131)”. No language limitation was considered and reference lists of all included articles and relevant reviews were evaluated for additional relevant articles. Two researchers (H.R and Z.M) searched the resources separately and coordinated with a third researcher (R.Z). Data abstraction was done with the following criteria; 1- Exclusion of review articles, editorials and meeting abstracts. 2- Inclusion of all controlled trials regardless of randomization process. Any article that could not be categorized according to title and abstract was reviewed completely, by the researchers. We divided activity selection methods in two groups: fixed dose or calculated dosimetry. We also classified the amount of I-131 prescribed to high dose or low dose. Primary outcome was the frequency of treatment success. Cure was defined as euthyroidism or permanent hypothyroidism, one year after treatment. Hypothyroidism was defined in all articles as high serum TSH and low serum T4 level. Euthyroidism was defined as normal TSH and normal serum T4 and serum T3. Hyperthyroidism was defined as suppressed TSH in the presence of elevated T4 or T3. Subclinical hyperthyroidism was defined as suppressed TSH with normal serum T4 and T3 levels. Subclincal hypothyroidism was defined as elevated TSH with normal serum T4 and T3 level.

### 2.1. Statistical Analyses

Considering the heterogenous nature of the included studies, random effects model (DerSimonian and Laird method) was used to pool the data. Two effect sizes namely risk ratio and risk difference were used for pooling the data. For heterogeneity evaluation, Cochrane Q test and I^2^ index were used and P < 0.05 was considered statistically significant. Funnel plot as well as Egger’s regression intercept method was used for publication bias evaluation. All analyses were performed using Comprehensive Metaanalysis software (V 2.2.050, USA).

## 3. Results

**Figure 1.**
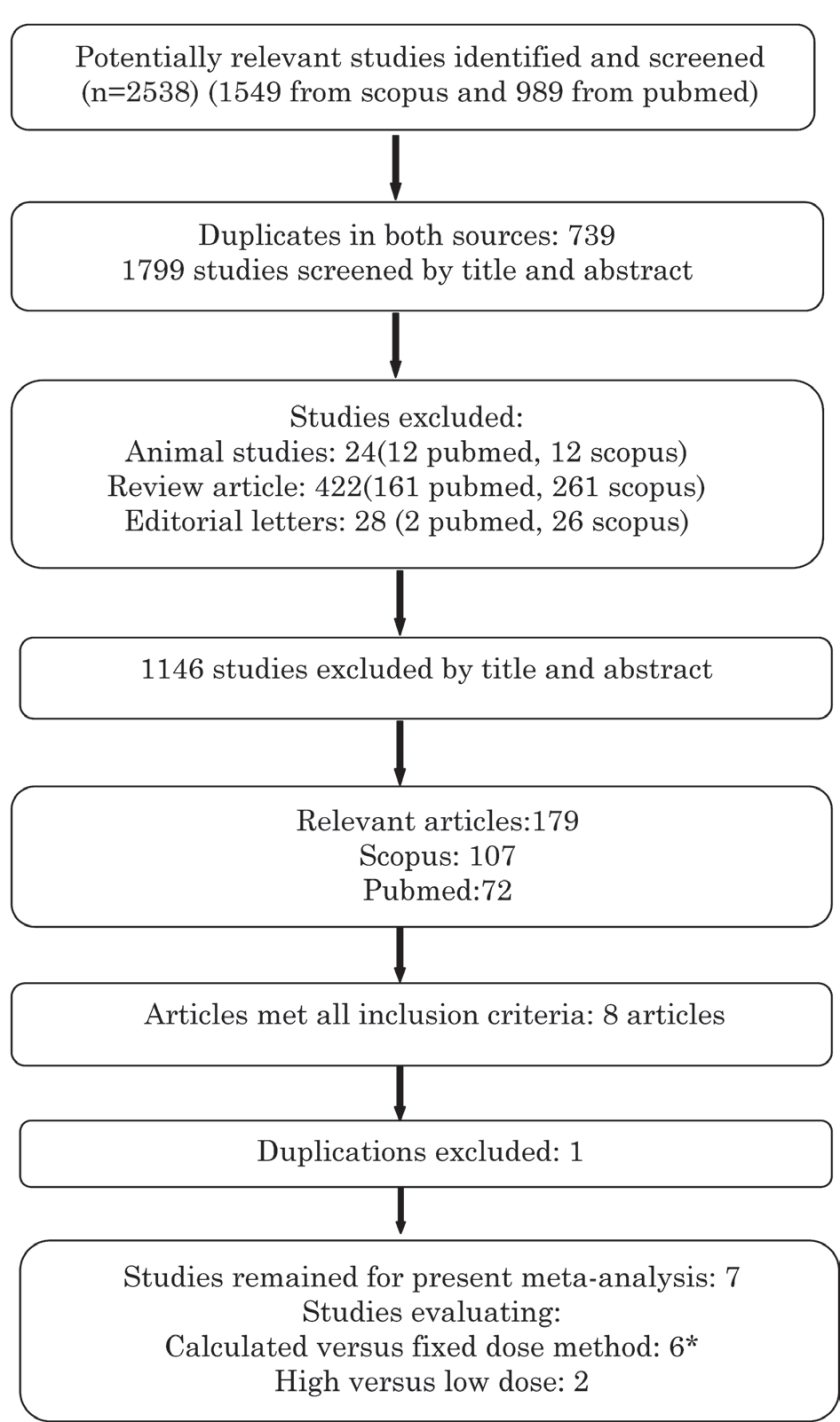
Flowchart of Study Inclusion in the Meta-Analysis

The literature search yielded 2538 articles (989 articles from Pubmed and 1549 articles from Scopus) from which 739 studies were indexed in both resources and 474 studies were congress abstracts, editorial letters, review articles, animal studies and children studies that were excluded (Figure 1). The remaining 1325 articles were evaluated by title and abstract and 1146 studies were irrelevant and discarded. Abstracts of all other articles (n = 179) were evaluated and any article reporting I-131 treatment in two or more groups was fully reviewed. The full text of all possibly relevant articles was obtained. Two randomized and three non-randomized clinical trials were considered for full review; two extra studies were found after searching the references of included trials. Finally seven articles (Two randomized and five non-randomized trials) including 669 patients with TNG met the eligibility criteria for analysis (Table 1).

The analysis was performed for comparison of FLD versus FHD regimens as well as calculated versus fixed dose (regardless of low or high) regimens. It should be noted that in one study, patients with single toxic adenoma were randomized into four groups and both calculated versus fixed dose and low versus high dose of I-131 were compared (5). Data from each part of this study was entered separately. In three studies only multinodular toxic goiters were included (12-14). Three studies comparing fixed dose (FD) versus calculated dose (CD), included both Graves’ disease and TNG (4, 15, 16). In recent studies we extracted data for patients with toxic multinodular goiter and only analyzed that part of the trial. Only two trials compared FHD and FLD of I-131 treatment (5, 12). In other studies the calculated and fixed dose methods of radioiodine therapy were compared. Cure of hyperthyroidism was defined as hypothyroidism or euthyroidism one year after therapy. Studies with calculated dosimetry method evaluated thyroid volume by palpation (5), ultrasonography (4, 16) or thyroid radioisotope scanning (13, 14). Appraisal of the articles (Table 2) was done using Oxford University table for critical appraisal for therapy studies (http://www.cebm.net/index.aspx?o= 1097 Accessed Nov 2012), separately, by two physicians.

**Table 1.** Characteristics of Included Studies ^a^

First Author	Origin	Year Published	Study Design	Diagnosis	Mean Follow up Months, Range	Number of Patients	Dosimetry Methods	Method of Thyroid Weight Estimation	Mean activity (MBq)
**Zakavi et al. (**5**)**	Iran	2009	RCT	TA	14 (2-49)	97 cd/fd	ETWx DA/ 24hr RAIU	Palpation	388/692/ 481/832
**Jarlov et al. (**4**)**	Denmark	1995	RCT	GD,TMG	12	62/49 cd/fd	ETWx DA/ 24hr RAIU	US	316/324
**Kok et al. (**15**)**	Netherland	2000	Non-RCT	GD,TMG	12	68/42 cd/fd	ETW x DA/ 24hr RAIU	Palpation	774/624
**Khanna et al. (**14**)**	India	1996	Non-RCT	TMG	(46-58)	55/75 cd/fd	ETW x DA/ 24hr RAIU	Nuclear imaging	N/A
**Sonmez et al. (**12**)**	Turkey	2011	Non-RCT	TA,TMG	17	35/58 fl/fh	Fixed	NA	370/740
**Huysmans et al. (**13**)**	Netherland	1993	Non-RCT	TMG	62	58/45 cd/fl	ETWx DA/ 24hr RAIU	Nuclear imaging	470/371 ^b^
**Ustun et al. (**16**)**	Turkey	2005	Non-RCT	GD, TMG,TA	6-134(TA) 7-122(TMG)	47/26cd/fl	ETWx120uci/24RAIU	US	445/370

^a^ Abbreviations: cd, Calculated dose; DA, dose administered; ETW, Estimated thyroid weight; fd, Fixed dose; fh, Fixed high dose; fl, Fixed low dose; RAIU, Radioactive Iodine uptake; RCT, randomized controlled trial; TA, toxic adenoma; GD, Graves’ Disease; TMG, toxic multinodular goiter; N/A, Not Available; US, Ultrasonography.

^b^ Normalized for thyroid weight.

**Table 2.** Quality Assessment of the Included Studies ^a,b^

First author	Randomization	Similarity Between Groups	Equal Treatment Apart From I-131	Lost to Follow Up
**Sonmez (**12**)**	No	no	no	0
**Huysmans (**3**)**	No	no	no	1.7
**Khanna (**14**)**	No	no	no	N/A
**Zakavi (**5**)**	Yes	yes	yes	37.1
**Jarlov (**4**)**	Yes	no	no	26.3
**Kok (**15**)**	No	no	yes	12.1
**Ustun (**16**)**	No	no	yes	N/A

^a^ Abbreviation: N/A, Not available.

^b^ Data are presented as %.

### 3.1. Calculated Versus Fixed Dose Method

**Figure 2.**
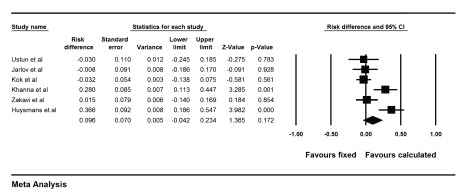
Forest Plot of Risk Difference of Cure of Hyperthyroidism in Different Studies Comparing Fixed Versus Calculated I-131 Dose The black squares are risk differences of individual studies and their sizes correspond to the sample size. Lines crossing each square represent 95% CI. The black diamond is the pooled risk difference of the included studies.

**Figure 3.**
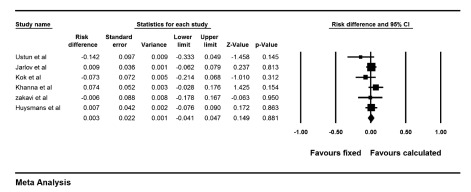
Forest Plot of Risk Difference Between Fixed and Calculated Methods Regarding Permanent Hypothyroidism As it can be seen there is no significant difference between these two techniques on permanent hypothyroidism.

The risk ratio of cure of hyperthyroidism was 1.17 (0.95-1.45) for CD versus FD method with a risk difference of 9.6% (-4%-23.4%), P = 0.17 (Figure 2). This means that patients treated with CD responded to I-131 therapy 9.6% points more than patients treated with the FD method. Cochrane Q test showed significant heterogeneity between the studies (I^2 ^= 77.2%, P = 0.001.). Funnel plot of the analysis showed minimal asymmetry. Also Egger’s regression intercept was -2.7 with p value of 0.09 concordant with funnel plot findings. Two studies used very high dose in calculated group on one hand and multiple small fixed doses (150 MBq) on the other hand (13, 14). Excluding these two studies, risk ratio was 0.98 (0.89-1.07, P = 0.6) for CD versus FD method with a risk difference of -1.7% (-9% -6%, P = 0.6). This means that cure of hypothyroidism was 1.7% points less for the CD method compared to the FD strategy. Permanent hypothyroidism was analyzed for CD versus FD methods and the results showed a risk ratio of 0.87 (0.53-1.42) and a risk difference of 0.3% (-4%- 5%, P = 0.8). This indicates that patients treated with calculated method have 0.3% points less permanent hypothyroidism than patients treated with fixed dose. Forest plot of pooled risk difference is shown in Figure 3.

### 3.2. High Dose Versus Low Dose Method

**Figure 4.**
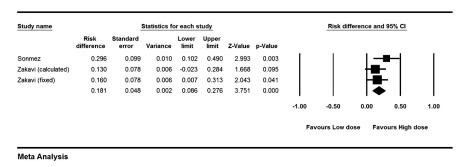
Forest Plot of Risk Difference of Cure of Hyperthyroidism in Different Studies Comparing High Versus Low Dose of Radio-Iodine The high dose method results in significantly higher number of responses compared to the low dose method.

**Figure 5.**
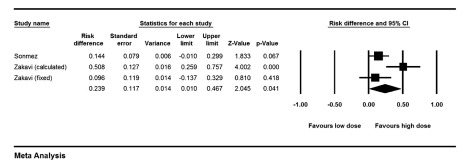
Forest Plot of Risk Difference of Permanent Hypothyroidism in Different Studies Comparing High Versus Low Dose of Radio-iodine The high dose protocols result in 23.9% more permanent hypothyroidism.

The risk ratio for cure of hyperthyroidism was 1.22 (1.06-1.41) comparing high dose (HD) versus low dose (LD) methods (P = 0.006). Risk difference analysis showed that patients treated with HD were cured 18.1% (8%-27.6%) points more than patients treated with LD of I-131 (P < 0.001), (Figure 4). Cochrane Q test failed to show any heterogeneity between studies (P = 0.39). Looking only at FHD versus FLD group, risk difference increased 21.5% points in high versus low dose protocols (95% CI: 0.08-0.34, P = 0.001). The risk ratio of permanent hypothyroidism was 2.40 (1.36-4.21) for HD versus LD (P = 0.002). The risk difference for development of hypothyroidism between patients treated with HD versus LD of I-131 was 23.9% (1%-46.7%, P = 0.041), (Figure 5). Again, analysis of FHD versus FLD protocols, yielded in a risk difference of 13% (0.1-25.8%) points in favor of high dose (P = 0.04).

## 4. Conclusions

In the present meta-analysis, treatment of hyperthyroidism by I-131 using fixed or calculated dose methods as well as fixed low versus fixed high dose methods in patients with TNG was compared. Although different methods yielded in successful treatment in the majority of patients with hyperthyroidism, our study showed more successful therapy for CD compared to FD method. Interestingly, permanent hypothyroidism was not significantly different between calculated and fixed dose regimens. Comparing low and high dose I-131 protocols, HD regimen, treated patients 18.1% more than LD regimen. However permanent hypothyroidism was also 23.9% more common in HD regimen. We used random effects model for pooling the data due to clinical heterogeneity among the studies, as mentioned above.

There was heterogeneity among studies regarding definition of low or high dose either in fixed protocols or calculated methods. The low dose ranged from 148 MBq to 555 MBq in fixed methods and from 1.23 MBq/g to 4.4 MBq/g in calculated dose protocols. The high dose ranged from 600-1200 MBq in fixed methods and 5.5-7.4 MBq/g in calculated dose protocols. Not all investigators used a defined fixed dose of I-131,some authors used different doses depending on the size of thyroid with increasing dose in larger volumes (17). One study used different doses for single nodular goiter and multinodular goiter (4). Even more complicating, one author used multiple small doses of I-131 in 6 months intervals (13). Also, method of determination of thyroid volume was not uniform among the studies. Anti-thyroid drugs had been discontinued in patients from 2-7 days before I-131 therapy in different studies. Radioactive iodine uptake (RAIU) was measured in all studies except one which applied fixed dose without measurements of RAIU (12).

de Rooij et al. in a meta-analysis of patients with hyperthyroidism, including both nodular and diffuse goiters, found no significant difference between calculated and fixed dose regimens (11). The advantage of our study compared to previous meta-analysis was that new studies were added and we analyzed studies of nodular goiters only and excluded diffuse goiters.

Considering total administered dose, we have lower dose in calculated methods compared to fixed dose methods in two studies (465.3 vs 545.6 MBq) and higher dose in calculated methods in another two studies (609.5 vs 497 MBq). Two other studies compared very high dose in calculated group with multiple fixed small doses (13, 14). Overall the mean administered dose in calculated method was 523.0 ±199.1 MBq and in fixed dose method was 526.0 ± 206.4 MBq. Considering higher response rate in calculated protocols compared to fixed dose method, this finding suggests that the calculated method may have the potential to treat more patients compared to fixed dose methods with same amount of I-131 administration. This is not surprising as thyroid dose is more scientifically determined in calculated method compared to the fixed method (18). The calculated method may be more acceptable considering as low as reasonably achievable (ALARA) principle and an increasing desire for lowering annual dose of the general population.

The goal of treatment of hyperthyroidism is to bring patients to the euthyroid state (19), however after I-131 therapy a significant number of patients become hypothyroid. Hypothyroidism may be transient (usually 2-4 months after therapy) or permanent. In young patients, permanent hypothyroidism is associated with long-term replacement therapy with thyroid hormones with its consequences on quality of life. Therefore optimal I-131 dose should also be associated with low incidence of permanent hypothyroidism. In our analysis, permanent hypothyroidism was 23.9% higher in FHD regimens compared to low dose strategies although cure rate was only 18.1% more in high dose methods. As a result, permanent hypothyroidism should always be considered in patients when treated with high dose of I-131 and may be more acceptable in treatment of old patients. Interestingly, in the calculated method, permanent hypothyroidism was only 0.3% more frequent compared to fixed dose regimens while cure rate was 9.6% higher in CD compared to FD strategies.

The trials were from different countries and although there was some heterogenisity in methodology in different studies, the result of this study can be generalized for other areas provided that randomization was observed. Using odds ratio may interfere with generalizability of the meta-analysis, thus we used risk difference and relative risk in our analysis (20).

Heterogeneity is the main shortcoming of meta-analysis of I-131 therapy in patients with hyperthyroidism. Absence of consensus either on high dose or low dose definition or thyroid volume determination methods as well as ignoring biologic half life of I-131 and non randomized grouping of the patients in different arms are among the main shortcomings of the studies in this field. Follow up of patients in this study was limited to one year, which may be considered as another limitation. We need large multicenter randomized trials to draw a robust conclusion.

Our study suggests that calculated method may be more appropriate for I-131 therapy of patients with TNG compared to fixed dose methods. Also higher doses are associated with more response, as well as more permanent hypothyroidism.
